# Multiple expression cassette exchange via TP901‐1, R4, and Bxb1 integrase systems on a mouse artificial chromosome

**DOI:** 10.1002/2211-5463.12169

**Published:** 2017-01-28

**Authors:** Kosuke Tomimatsu, Kenji Kokura, Tadashi Nishida, Yuki Yoshimura, Yasuhiro Kazuki, Masashi Narita, Mitsuo Oshimura, Tetsuya Ohbayashi

**Affiliations:** ^1^ Research Center for Bioscience and TechnologyTottori UniversityYonagoJapan; ^2^ Japan Society for the Promotion of ScienceTokyoJapan; ^3^ Chromosome Engineering Research CenterTottori UniversityYonagoJapan; ^4^ Division of Human Genome ScienceDepartment of Molecular and Cellular BiologySchool of Life SciencesFaculty of MedicineTottori UniversityYonagoJapan; ^5^ Department of Biomedical ScienceInstitute of Regenerative Medicine and BiofunctionGraduate School of Medical SciencesTottori UniversityYonagoJapan; ^6^ Central Institute for Experimental AnimalsKawasakiJapan; ^7^ Cancer Research UK Cambridge InstituteLi Ka Shing CentreUniversity of CambridgeUK

**Keywords:** fluorescent protein, integrase, lineage‐tracing reporter, luciferase, mouse artificial chromosome

## Abstract

The site‐specific excision of a target DNA sequence for genetic knockout or lineage tracing is a powerful tool for investigating biological systems. Currently, site‐specific recombinases (SSRs), such as Cre or Flp recombination target cassettes, have been successfully excised or inverted by a single SSR to regulate transgene expression. However, the use of a single SSR might restrict the complex control of gene expression. This study investigated the potential for expanding the multiple regulation of transgenes using three different integrase systems (TP901‐1, R4, and Bxb1). We designed three excision cassettes that expressed luciferase, where the luciferase expression could be exchanged to a fluorescent protein by site‐specific recombination. Individual cassettes that could be regulated independently by a different integrase were connected in tandem and inserted into a mouse artificial chromosome (MAC) vector in Chinese hamster ovary cells. The transient expression of an integrase caused the targeted luciferase activity to be lost and fluorescence was activated. Additionally, the integrase system enabled the specific excision of targeted DNA sequences without cross‐reaction with the other recombination targets. These results suggest that the combined use of these integrase systems in a defined locus on a MAC vector permits the multiple regulation of transgene expression and might contribute to genomic or cell engineering.

**Abbreviations**
CLuc: *Cypridina* luciferin 2‐monooxygenase
EGFP: enhanced GFP
ELuc: enhanced beetle luciferase
FISH: fluorescence *in situ* hybridization
GFP: green fluorescent protein
GLuc: *Gaussia* luciferase
HPRT: hypoxanthine phosphoribosyltransferase
MAC: mouse artificial chromosome
MEEVS: multiple expression cassette exchange via site‐specific recombinases
OFP: orange fluorescent protein
PAC: P1 artificial chromosome
pCAG: CAG promoter
RE: restriction enzyme
RFP: red fluorescent protein
SSR: site‐specific recombinase

Site‐specific recombination has been used *in vitro* and *in vivo* to manipulate predesigned DNA sequences, including the introduction of foreign gene cassettes, generation of conditional knockout models, and lineage tracing 1, 2, 3, 4, 5, 6. These reactions are designed using site‐specific recombinase (SSR) systems. Cre‐loxP from bacteriophage P1 7, 8 and Flp‐FRT from *Saccharomyces cerevisiae* 9 are the most widely used systems. These Cre or Flp recombinases recognize specific nucleotide sequences (loxP or FRT), and perform a reversible catalytic reaction between two loxP or FRT sites, respectively. Other mechanistically distinct recombinases include ΦC31 integrase derived from the *Streptomyces* phage, ΦC31 10. The integrase catalyzes recombination between attP and attB sites. However, the enzyme does not catalyze the resulting attL and attR sequences 11. This one‐way reaction has been successfully applied for site‐directed insertion and in gene targeting studies 12, 13. Similarly, several integrases such as TP901‐1 from *Lactococcus lactis* 14, R4 from *Streptomyces parvulus* 15, and Bxb1 from *Mycobacterium smegmatis* 16 have been evaluated for their use in mammalian cells 17, 18, 19, 20. Target DNA sequences can be inserted, excised, inverted, and translocated, and are subject to cassette exchange using these recombinase or integrase systems. The combined or individual use of these systems provides a wide range of control for gene expression 21, 22.

Lineage tracing describes the detection of the progeny of cells that are marked with the aim of following cell movements and/or cell fate. In mammalian cells, the Cre‐loxP system is widely used to mark cells for lineage‐tracing experiments 23. A reporter construct containing transcription stop sequences (polyadenylation signals) flanked by loxP sequences is placed under the control of a ubiquitous promoter, and the loxP‐flanked transcription stop sequence is followed by a reporter gene, such as LacZ, enhanced green fluorescent protein (EGFP), or tandem‐dimer red fluorescent protein (RFP) 24, 25, 26, 27. The temporal expression of Cre recombinase excises the stop sequences between loxP sites resulting in the expression of reporter genes under control of the same ubiquitous promoter 23. A similar lineage‐tracing reporter system using Flp‐FRT is also commonly used in *Drosophila melanogaster* and mammals 28, 29, 30. Recently, ΦC31 integrase was employed as another option for lineage‐tracing reporter systems 31, 32; however, Grandchamp *et al*. 33 reported that ΦC31 integrase might induce DNA damage. Therefore, the current study investigated the potential of other integrases, TP901‐1, R4, and Bxb1.

The conventional method of producing transgenic cells using plasmid transfection or retrovirus infection frequently leads to clonal variability. Random integration of a transgene into the host genome sometimes causes the disruption of host genes and expression of the transgene can be restricted by this positional effect 34, 35. To overcome these problems, human artificial chromosome (HAC) vectors were produced by deleting all the endogenous genes in human chromosome 21 36, 37. HAC vectors replicate and are segregated in a manner similar to natural chromosomes, and independently from the host genome in mammalian cells. Transgene expression on HAC vectors is stable and long‐lasting 38, 39. We recently used mouse chromosome 11 to produce a mouse artificial chromosome (MAC) vector for the stable maintenance of transgenes in mouse cells 40, 41. In this study, we used the MAC vector to investigate the feasibility of the transgene regulation system because other open chromatin loci, such as *Rosa26* and *AAVS1,* are often used for other genomic manipulation purposes 42, 43, 44, 45.

Luciferases are often used as reporters to monitor gene expression because of their high sensitivity and wide range of linear responses. Although firefly luciferase is most commonly used and utilizes luciferin as a substrate, enhanced beetle luciferase (ELuc) has higher catalytic properties and thermostability than firefly luciferase 46. Recently, other luciferases such as *Gaussia* luciferase (GLuc) and *Cypridina* luciferin 2‐monooxygenase (CLuc) were developed 47, 48. These two luciferases are secreted into the extracellular environment (blood, urine, or culture media) when expressed in cells. Because ELuc, GLuc, and CLuc utilize different substrates (luciferin, coelenterazine, and vargulin, respectively), each luciferase activity can be separated 49. We used these luciferases to monitor the precise excision of transcription stop sequences for the reporter system after the expression of integrases.

Here, we report the construction of a multiple expression cassette exchange via a SSRs (MEEVS) system using three distinct integrases, TP901‐1, R4, and Bxb1, for complex transgene manipulation. The efficiency and specificity of site‐specific recombination at the defined locus on the MAC vector were evaluated in mammalian cells.

## Materials and methods

### Cell culture

Hypoxanthine phosphoribosyltransferase (HPRT)‐deficient Chinese hamster ovary [CHO (*hprt* ^−/−^)] cells carrying a MAC2 vector were cultured in Ham's F‐12 nutrient mixture (Invitrogen, Carlsbad, CA, USA) containing 100 U·mL^−1^ of penicillin (Invitrogen), 100 μg·mL^−1^ of streptomycin (Invitrogen), 10% fetal bovine serum (JRH Biosciences, Lenexa, KS, USA), and 200 μg·mL^−1^ hygromycin B (Wako, Osaka, Japan) at 37 °C in a humidified atmosphere comprising 5% CO_2_.

### Construction of the MEEVS reporter vector

The vectors were purchased from various companies: pEGFP‐N1 (Clontech, Palo Alto, CA USA), pELuc‐test (TOYOBO, Osaka, Japan), pGluc‐basic (New England Biolabs; NEB, Beverly, MA, USA), phmKO2‐MN1 (Kusabira Orange 2; Amalgaam, Tokyo, Japan), and pTurboFP635 (Evrogen, Moscow, Russia). pCX‐EGFP (pCAG‐EGFP) was kindly provided by M. Okabe (Osaka University, Osaka, Japan). pBluescript‐SK(‐)2 CLm (containing CLuc) was kindly provided by Y. Nakajima (National Institute of Advanced Industrial Science and Technology, Kagawa, Japan). pDEST was kindly provided by M. Hiratsuka (Tottori University, Tottori, Japan). Integrated DNA Technologies (Integrated DNA Technology; IDT, IA, USA) synthesized cassettes encompassing multiple cloning sites flanked by recombinational attachment sites in the context of the pIDT‐SMART‐KAN vector (Figs S1 and S2); pIDT‐L1mcsL4, pIDT‐R4mcsR3 (TP901PB, R4PB, and Bxb1PB), and pIDT‐L3mcsL2.

DNA fragments [CAG promoter, GLuc, CLuc, ELuc, GFP, orange fluorescent protein (OFP), and RFP] were prepared using restriction endonucleases appropriate to each vector (pCX‐EGFP, pGLuc‐basic, pBluescript‐SK(‐)2 CLm, pELuc‐test, pEGFP‐N1, phmKO2‐MN1, and pTurboFP635). The CAG DNA fragment was recombined into pIDT‐L1mcsL4 (Donor 1) using the restriction sites *Sal*I and *Eco*RI. GLuc, CLuc, and ELuc gene fragments were recombined using the appropriate restriction sites into pIDT‐R4mcsR3‐TP901PB (*Xho*I and *Bam*HI), pIDT‐R4mcsR3‐R4PB (*Eco*RI and *Bam*HI), and pIDT‐R4mcsR3‐Bxb1PB (*Eco*RI and *Bam*HI), respectively (Donor 2). GFP (EGFP), OFP (Kusabira Orange 2), and RFP (TurboFP635) gene fragments were recombined into pIDT‐L3mcsL2 (Donor 3). Donor vectors 1, 2, and 3 and the pDEST vector were assembled using a Multisite Gateway™ (Invitrogen) kit 50, 51 to create three kinds of integrase‐mediated luminescence/fluorescence variable reporter vectors. Before assembly, donor vectors were digested with *Hin*dIII and separated into the att‐flanked region and the vector backbone region. Donor 1, 2, and 3 fragments flanked by att sites and the pDEST vector were mixed with 2 μL of LR clonase II Plus enzyme mix (Invitrogen) and made up to 10 μL with TE buffer. The mixture was incubated at 25 °C for 16 h. After incubation, the enzyme was inactivated by treatment with Proteinase K for 10 min at 37 °C and the assembled vector was then transformed into competent Mach1 T1^R^ *Escherichia coli*. DNA products were purified using a general DNA preparation method from transformed *E. coli*. The three different reporter vectors were recombined in tandem into the same P1 artificial chromosome (PAC) carrying a loxP sequence and the 3′ HPRT sequence (pPAC‐MEEVS reporter) using restriction sites with compatible ends for *Nhe*I and *Avr*II.

### Transfection

CHO (*hprt* ^−/−^) cells containing MAC2 were plated at a density of 5 × 10^5^ in six‐well tissue culture plates. The next day, 3.5 μg of the pPAC‐MEEVS reporter vector carrying loxP and the 3′ HPRT vector, and 0.5 μg of the Cre recombinase expression plasmid pBS185 were transfected into CHO MAC2 cells using Lipofectamine 2000 (Invitrogen). CHO clones containing the correct reporter inserted into MAC2 by site‐specific recombination via Cre recombinase were selected using culture medium supplemented with HAT (100 μm hypoxanthine, 0.4 μm aminopterin, and 16 μm thymidine; Sigma, St. Louis, MO, USA).

### Fluorescence *in situ* hybridization (FISH)

FISH analysis of CHO cells was performed with either fixed metaphase or interphase nuclei using digoxigenin‐labeled (Roche, Basel, Switzerland) mouse Cot‐1 DNA (Invitrogen) and biotin‐labeled DNA from the pPAC‐MEEVS reporter, as described previously 52. Chromosomal DNA was counterstained with DAPI (Southern Biotech, Birmingham, AL, USA). The images were captured using an Axio ImagerZ2 fluorescence microscope (Carl Zeiss GmbH, Jena, Germany) and analyzed with the Isis FISH imaging system (MetaSystems, Altlussheim, Germany).

### Assay for integrase‐mediated recombination of MEEVS on MAC2

CHO cells containing the MEEVS‐reporter‐MAC2 were plated at a density of 1 × 10^5^ in 24‐well tissue culture plates 24 h before transfection. CHO cells were transfected with 0.5 μg of integrase expression plasmid pCMV‐TP901‐1, pCMV‐R4, and pCMV‐Bxb1, as previously described 53 using Lipofectamine 2000 (Invitrogen). Seventy‐two hours after transfection, flow cytometric analysis was performed with a Gallios flow cytometer (Beckman Coulter, Fullerton, CA, USA) to determine the ratio of cells expressing fluorescent proteins after site‐specific recombination via integrase. GFP (EGFP), OFP (Kusabira Orange 2), and RFP (TurboFP635) were excited with a 488‐nm argon laser, and detected with band‐pass filters of 525/40 nm, 575/30 nm, and 620/30 nm, respectively. flowjo software (Tree Star Inc., Ashland, OR, USA) was used for subsequent data analysis. Cell sorting was performed with MoFlo XDP (Beckman Coulter) equipped with a 488‐nm argon laser.

### Luciferase assay

CHO cells carrying the MEEVS‐reporter‐MAC2 were plated at a density of 2 × 10^5^ in 24‐well tissue culture plates and cultured for 48 h. The conditioned media and cells were separately collected and used to measure luciferase activity. Activities of GLuc and CLuc luciferase secreted into the supernatant were measured using a BioLux® *Gaussia* Luciferase Flex Assay Kit (NEB) and a BioLux® *Cypridina* Luciferase Assay Kit (NEB), respectively. Twenty microliter of various conditioned cell culture media were transferred to flat solid bottom and opaque‐walled white 96‐well plates (Nunc, Roskilde, Denmark). GLuc and CLuc bioluminescence values were measured immediately after the addition of 50 μL of substrate solution. Expression of ELuc in cells was measured with the Emerald Luc Luciferase Assay Reagent (TOYOBO). Cells were resuspended at a density of 1 × 10^5^ cells per 50 μL with PBS and transferred to flat solid bottom and opaque‐walled white 96‐well plates (Nunc). ELuc bioluminescence values were measured after the addition of 50 μL of assay reagent and incubation for 10 min. An Infinite F500 luminometer (Tecan, Crailsheim, Germany) was used to determine luciferase activity.

### Microscopic imaging

CHO cells were cultured on glass bottom dishes and fixed with 4% paraformaldehyde phosphate buffer solution (Wako). Washed cells were mounted with Dapi‐Fluoromount‐G™ (Southern Biotech). Images were captured with an LSM 710 microscope (Carl Zeiss GmbH). For four‐color imaging, DAPI was excited with a 405‐nm diode laser, GFP was excited with a 488 nm argon laser, OFP (Kusabira Orange 2) was excited with a 561‐nm DPSS laser, and RFP (TurboFP635) was excited with a 633‐nm HeNe laser. The emissions of each type of fluorescence were detected at the optimal wavelength region with a variable bandwidth filter for minimizing bleed‐through between colors.

## Results

### Construction of multiple expression cassette exchange via SSR reporters

**Figure 1:**
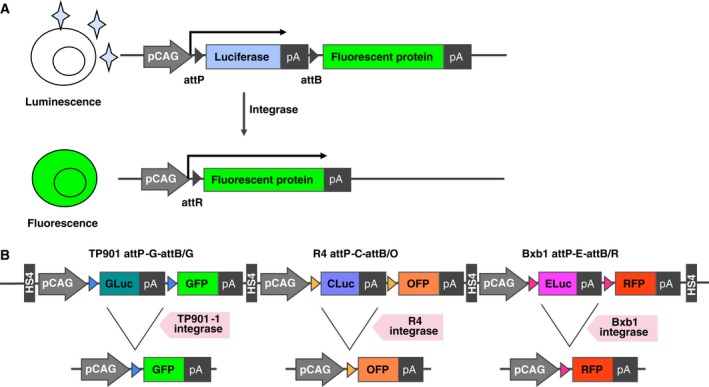
Schematic diagram of the MEEVS reporter unit. (A) Each unit of MEEVS reporter contains CAG promoter (pCAG), luciferase coding sequence with polyadenylation signal (pA) and fluorescent protein coding sequence. The initial MEEVS reporter unit expresses luciferase, but the fluorescent protein is not expressed. Because the luciferase expression cassette is flanked by specific integrase recognition sites (attP and attB), the luciferase cassette is excised upon expression of the specific integrase, and then the fluorescent protein cassette is expressed by pCAG. (B) GLuc, CLuc, and ELuc were used for luciferase, together with TP901, R4, and Bxb1 integrase recognition sites. GFP, OFP, and RFP were employed for fluorescent proteins in the MEEVS reporter.

We constructed a reporter system to evaluate the precise target DNA excision using three integrases, TP901‐1, R4, and Bxb1, which can be used for lineage tracing (Fig. 1). We designed a construct containing the luciferase gene with a transcription stop sequence (polyadenylation signal) between attP and attB sequences that are specific for each integrase, followed by a fluorescent reporter gene. For the ubiquitous promoter, we utilized a CAG promoter (pCAG). We intended that the luciferase gene between the integrase recognition sequences would be excised after the temporal expression of integrases, and then the fluorescent reporter would be expressed. We considered that a tandem array of reporter units for each integrase would help to evaluate comparatively the recombination rates among reporter units. To minimize the transcription interference of pCAG among reporter units, we added a DNase I hypersensitive 4 (HS4) site from chicken β‐globin 54, 55 between each reporter unit. Because this configuration of the construct enables the expression of more than one gene to be swapped using different integrases, we designated this platform as multiple expression cassette exchange via SSRs (MEEVS).

**Figure 2:**
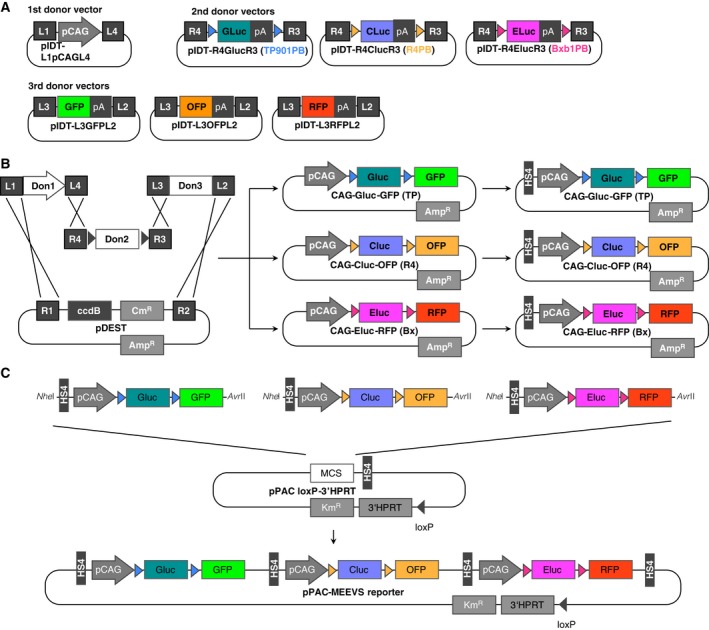
Construction of the MEEVS reporter on PAC vector. (A) Construction of donor vectors for components of MEEVS expression units. All donor vectors contain a pair of LR Clonase recognition sites (L1–L4 or R3/R4) for the Gateway system. The first donor vector contains CAG promoter (pCAG), and second donor vectors contain luciferase genes (Gluc, CLuc, or ELuc) together with a polyadenylation signal (pA) that is flanked by integrase recognition sites for TP901, R4, or Bxb1 (colored arrowheads). The third vectors contain fluorescent protein expression cassettes (GFP, OFP, or RFP followed by pA). (B) Assembling the donor vectors on pDEST vector using the Gateway system. Each MEEVS reporter unit was produced using LR Clonase that contained pCAG, luciferase between the integrase recognition sites and a fluorescent protein expression cassette. Then, an insulator sequence, DNase I hypersensitive 4 (HS4) site of chicken β‐globin, was added to each of MEEVS reporter unit. (C) Assembling MEEVS reporter units on a PAC vector. Using a *Nhe*I restriction enzyme site, each MEEVS reporter unit was sequentially inserted into the PAC vector that comprises an HS4 insulator, loxP sequence, and 3′ part of the *HPRT* gene, which are required for the subsequent transfer of the resulting pPAC‐MEEVS reporter into mouse artificial chromosome (MAC).

**Figure 3:**
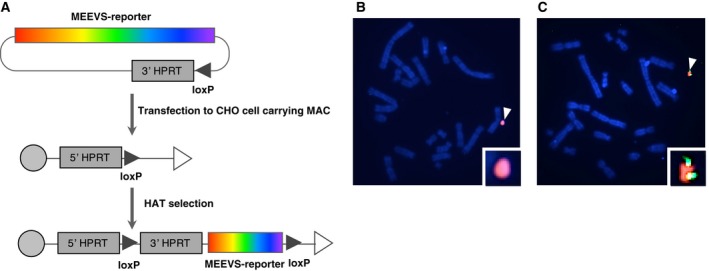
Site‐specific insertion of the MEEVS reporter into a mouse artificial chromosome (MAC). (A) pPAC‐MEEVS reporter and Cre recombinase expression plasmid were cotransfected into CHO cells (*hprt* ^−/−^) carrying a MAC vector. The MEEVS reporter was integrated into the MAC and regenerated a functional *HPRT* gene. (B) and (C) FISH analysis of CHO carrying MAC vectors. The white arrowhead and red signals (mouse Cot‐1 probe) indicate MAC vector; green signals indicate MEEVS reporter (probe of MEEVS vector labeled with FITC). CHO cells harboring empty MAC vector (B) or MEEVS reporter‐containing MAC (C) were used.

To construct a MEEVS cassette, we initially prepared three types of donor vectors for efficient cloning using the Gateway system. The first donor vectors contained the restriction enzyme (RE) sites *Nhe*I and pCAG flanked by attL1 and attL4 sites for Gateway LR clonase (Fig. 2). The second donor vectors had different luciferase genes between the distinct integrase recognition sites TP901, R4, or Bxb1; for each integrase, we inserted GLuc, CLuc, or ELuc, respectively (Fig. 2). The third vectors comprised fluorescent proteins (GFP, OFP, or RFP) followed by an *Avr*II RE site. These donor vectors and pDEST vectors were mixed in the presence of LR clonase enzyme to generate TP901‐1, R4, and Bxb1 operational vectors (MEEVS reporter units). HS4 sites were added into the 5′ end of pCAG of each reporter unit vector. Subsequently, the reporter inserts were assembled and ligated onto a PAC vector that contained a single loxP site and the 3′ part of mouse *Hprt* (Fig. 2). By using the Cre‐loxP system, the MEEVS platform was finally transferred into MAC2 that had the 5′ part of mouse *Hprt* and a loxP site in CHO (*hprt* ^−/−^) cells (Fig. 3). We cultured and selected CHO cells with HAT medium, and picked up several clones. We performed FISH analyses to identify clones that contained the correct MAC vector without insertions of the reporter in the host genome.

### Integrase‐mediated expression cassette exchange on a MAC vector

**Figure 4:**
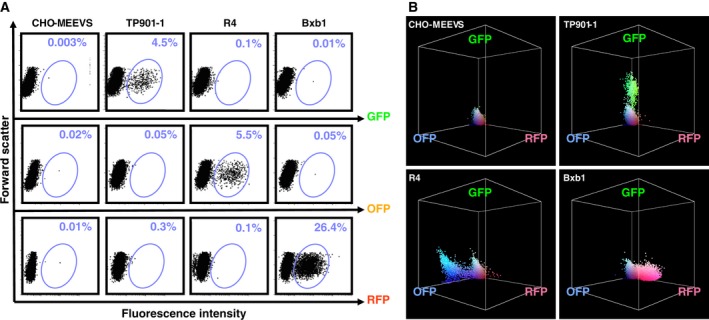
Site‐specific gene excision for multiple recombination targets. (A) Flow cytometric analysis of CHO‐MEEVS cells transfected with 2 μg of integrase (TP901‐1, R4, or Bxb1) at 72 h after transfection. The blue circle indicates a population of fluorescent protein‐positive cells (total of 30 000 counted cells). (B) Three‐dimensional analysis of the specificity of the integrase reactions. Integrase transfected cells were analyzed by flow cytometry. Cells were counted until 10 000 single fluorescent‐positive cells were identified and the compensated data were plotted on three axes of fluorescence intensity (GFP, OFP and RFP).

**Figure 5:**
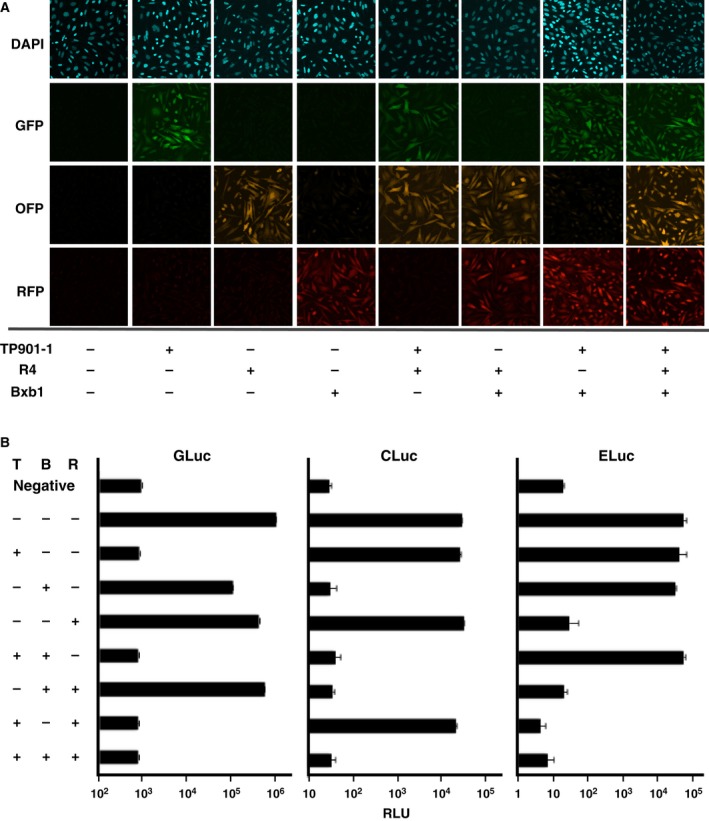
Microscopic and luciferase analysis of CHO‐MEEVS cells. (A) CHO‐MEEVS cells were transfected with integrase expression plasmids, purified by flow cytometry, then the cells were observed by fluorescent microscopy. Each florescent protein (GFP, OFP, and RFP) was observed using an appropriate filter set. Combinations of transfected integrases are shown by +. (B) Luciferase assays of CHO‐MEEVS. For GLuc and CLuc assays, conditioned culture media of cells were used. For ELuc assays, the cells were resuspended in PBS and mixed with assay reagent, which contained cell lysis buffer and substrates. Combinations of transfected integrases are shown by +. CHO cells harboring empty MAC vector were used as negative controls. Three independent samples were used to determine the luciferase activities, and mean values with standard deviation are shown. T; TP901‐1, R; R4, B; Bxb1, RLU; relative light unit.

The CHO cells described above (hereafter, CHO‐MEEVS cells) showed no detectable fluorescence from GFP, OFP, or RFP (Fig. 4A, CHO‐MEEVS). We transfected cells with each integrase expression plasmid and performed flow cytometry analysis. When TP901‐1, R4, or Bxb1 integrases were expressed in CHO‐MEEVS cells, only one of GFP, OFP, or RFP was observed in the respective transfected cell line (Fig. 4). The specific expression of these fluorescent proteins was confirmed under microscopy using flow‐sorted cells (Fig. 5A). We observed that Bxb1 yielded a higher ratio of fluorescence‐positive cells compared with TP901‐1 or R4, suggesting that Bxb1 catalyzed recombination more efficiently than TP901‐1 and R4 in CHO‐MEEVS cells (Fig. 4). To estimate the transfection efficiency of CHO cells, we expressed EGFP using the pCAG‐EGFP plasmid instead of an integrase. Approximately, 30% of the cells were GFP positive (Table 1). Therefore, the recombination efficiency of Bxb1 was considered greater than 85%, and for TP901‐1 or R4, it was 15–25% (Table 1). This is consistent with other reports of integrase activity in the human HT1080 cell line 56, mouse ES cells 41, and in CHO cells using HAC 53. We also measured the luciferase activities of GLuc, CLuc, and ELuc. All luciferase activities were detected using distinct substrates in initial CHO‐MEEVS cells (Fig. 5B). The activity of GLuc, CLuc, or ELuc was lost upon transfection of TP901‐1, R4, or Bxb1 integrases, respectively. The diminished luciferase activity verified the substrate specificity and negligible cross‐reactivity in the assay. Furthermore, the expression of GLuc, CLuc, and ELuc were mutually exclusive to GFP, OFP, and RFP implying precise recombination between the recognition sites for the specific integrases on the MAC vector in CHO cells (Fig. 5).

**Table 1:** Percentage of fluorescent protein‐positive cells. pCAG, CAG promoter; EGFP, enhanced green fluorescent protein

Transfected vector	Trial		
	1	2	3
pCAG‐EGFP	29.9%	27.1%	27.3%
TP901‐1 integrase	4.5% (15.1%)	4.6% (17.0%)	4.1% (15.0%)
R4 integrase	5.5% (18.4%)	6.7% (24.7%)	5.9% (21.6%)
Bxb1 integrase	26.4% (88.3%)	24.2% (89.3%)	23.2% (85.0%)

Percentage in bracket indicates normalized number by transfection efficiency of pCAG‐EGFP in each trial.

Next, we tested whether a combination of integrases could also catalyze appropriate target sites without disturbing each other. Cotransfection of two of integrases and subsequent flow‐sorting generated cells expressing two fluorescent proteins resulted in the decreased activity of two of luciferases accordingly. When we expressed all the integrases, CHO‐MEEVS cells exhibited the expression of GFP, OFP, and RFP fluorescence but lost luciferase activities as expected. These results indicated that the simultaneous expression of these integrases could function separately toward their target sequences on a MAC vector in CHO cells.

## Discussion

In the present study, we constructed a reporter system to evaluate the precise target DNA excision for TP901‐1, R4, and Bxb1 integrases, which can be used for lineage tracing. Single or combinational expression of TP901‐1, R4, and Bxb1 enabled the marking of cells by up to eight different expression patterns of fluorescent proteins using the MEEVS reporter. In lineage‐tracing experiments, the expression of a single SSR driven by a single promoter was sometimes insufficient to mark the cells of interest because single promoter activity is not always specific to the cells. To increase the resolution of genetic lineage tracing, Split Cre, Co‐In‐Cre, and Co‐Driver systems, in which Cre recombinase activity is regulated by two different promoters, have been developed using single lineage‐tracing reporter 57, 58. The MEEVS reporter described here could be another option to follow precise cell lineages conferring other layers of reporter units.

Differences in recombination efficiency among distinct integrases are sometimes problematic for practical use. We performed a comparative assessment of site‐specific DNA excision by the transfection of integrase expression plasmids in CHO cells carrying the MEEVS reporter. Differences in recombination rates were observed for each integrase, despite use of the same promoter. In particular, the Bxb1 integrase system showed consistently higher recombination efficiency than the TP901‐1 and R4 integrases.

This study demonstrated the feasibility of combinations of luminescent/fluorescent reporters, which indicated the precise excision of stop sequences occurred in the MEEVS reporter. We confirmed the separation of each luciferase signal, even that highly expressed by pCAG. To prepare positive or negative controls for the later flow cytometry experiments, fluorescent microscopy, and luciferase analyses, we purified the repertoire of CHO cells expressing all combinations of the reporters by flow sorting. These cells were used as controls for microscopic analyses and luciferase assays (Fig. 5).

We also demonstrated that the systematic construction of these operational transgenes was regulated by multiple integrases. We designed targeted DNA excision modules using gateway technology to facilitate the construction of each unit (operational vector) and these units were connected in tandem using compatible RE sites, such as *Nhe*I, *Avr*II, *Xba*I, and *Spe*I (Fig. 2 and Fig. S1). Integrase systems other than the three described here could also be used by inserting their recognition sites into a second donor vector for additional multiple manipulation of transgenes (Fig. 2 and Fig. S1). This configuration of the MEEVS system can be used for both lineage‐tracing reporters and the conditional expression of genes of interest instead of fluorescent reporters (Fig. S1). Because tissue‐specific promoters can also be used in first donor vectors other than pCAG, the expression of genes of interest might be controlled in a more complex manner.

In an example of this methodology in an *in vivo* mouse study, lineage trace experiments were performed by crossing a mouse strain carrying an operational allele and a mouse strain carrying a driver allele, each of which were knocked into a defined locus, such as *Rosa26,* as well as tissue‐specific genes 24, 59, 60. The transgene, including the recombination cassette on the operational allele, was then modified by the SSR from the driver allele when the cell retained both alleles. We used a MAC vector as a defined genomic locus for the operational allele to ensure accurate copy control and stable expression 37, and therefore, SSR and other transgenes can be integrated into endogenous genome loci.

Recently, our group reported that plant (*Arabidopsis*) chromosomes could be maintained in a human cell background 61. This suggests that plant chromosomes might serve as material for a shuttle chromosome vector between animals and plants if the plant chromosome can be transferred back into plant cells. Because SSRs have been used in plant cells as well as in yeast, flies, and mammals for genomic manipulation 22, 62, 63, 64, 65, the MEEVS system on an artificial chromosome may be used for a broad range of organisms including plants in the future. We used the MEEVS system for reporter genes (luciferases and fluorescent proteins) in this study, but the system can also be applied to other genes of interest including genome‐editing tools (zinc finger nuclease, TALEN, or CRISPR/CAS9) to investigate phenotypes of loss of function and/or gain of function 66. Collectively, we constructed a MEEVS platform that permits the complex expression of genes of interest in up to eight configurations using three distinct integrases.

## Conclusions

We generated a reporter system to evaluate the precise target DNA excision for TP901‐1, R4, and Bxb1 integrases, which can be used for lineage tracing. In the reporter system, luciferase expressions were exchanged to fluorescent proteins in response to the transient expression of specific integrases in CHO cells. Because we used three distinct luciferases (GLuc, CLuc, and ELuc) and fluorescent proteins (GFP, OFP, and RFP) for each reporter unit of the integrase, we could mark cells with up to eight different expression patterns of fluorescent proteins. The reporter system was inserted into a MAC vector, which harbors an alternative defined locus for the stable expression of reporters other than endogenous genomic loci, such as *Rosa26* and *AAVS1*. The MEEVS platform described here may lead to complex genomic manipulations not only in tissue culture cells but also in yeast, animals, and plants, by using combinations of SSRs, artificial chromosomes, and other genome‐editing tools 61, 66, 67, 68.

## Author contributions

TO conceived and supervised the study; MO and MN designed experiments; KT, TN, and YY performed experiments; KT, KK, and TO wrote the paper; YK provided the samples and technical support.

## Supporting information


**Fig. S1.** Schematic of MEEVS composition and vector construction.
**Fig. S2.** Nucleotide sequences of synthesized DNA fragments that were inserted into the donor vectors shown in Fig. S1.
