# Supplementary material for: Multiple expression cassette exchange via TP901‐1, R4, and Bxb1 integrase systems on a mouse artificial chromosome
Source: FEBS Open Bio. 2017 Jan 28;7(3):306–17. doi: 10.1002/2211-5463.12169 (PMC5337897; doi:10.1002/2211-5463.12169)
Supplement: Supplementary file 1 — Fig. S1. Schematic of MEEVS composition and vector construction. Fig. S2. Nucleotide sequences of synthesized DNA fragments that were inserted into the donor vectors shown in Fig. S1. [file FEB4-7-306-s001.docx]

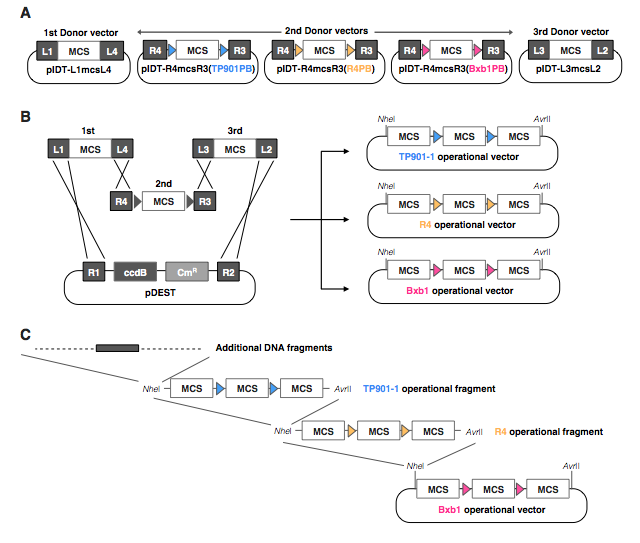


Supplementary Figure S1. Schematic of MEEVS composition and vector construction.

(A) The first donor vector contains attL1-MCS-attL4. The second donor vector contains attR4 and an MCS flanked by specific-integrase recognition sequences: attB and attP (color triangles) and attR3 for LR cloning. The third donor vector contains attL3-MCS-attL2. (B) Donor vectors were assembled by LR Clonase; operational vectors with one pair of attachment sites recognized by a specific integrase were produced. (C) These operational vectors were sequentially combined with restriction digestion sites *Nhe*I and *Avr*II present at both ends of the operational fragment.

pIDT-L1mcsL4

AGATCTAAGCTTAGTCTTAAGCTCGGGCCCCAAATAATGATTTTATTTTGACTGATAGTGACCTGTTCGTTGCAACAAATTGATGAGCAATGCTTTTTTATAATGCCAACTTTGTACAAAAAAGCAGGCTAGCTCACTCATTGTCGACCCGGGATCCGAATTCATGCAGGACCCAACTTTTCTATACAAAGTTGGCATTATAAGAAAGCATTGCTTATCAATTTGTTGCAACGAACAGGTCACTATCAGTCAAAATAAAATCATTATTTGCCATCCAGCTGCAGGGCCCAAGCTT

pIDT-R4mcsR3 (TP901PB)

AGATCTGCTAGCAAGCTTGGGCCCCTACAGGTCACTAATACCATCTAAGTAGTTGATTCATAGTGACTGGATATGTTGTGTTTTACAGTATTATGTAGTCTGTTTTTTATGCAAAATCTAATTTAATATATTGATATTTATATCATTTTACGTTTCTCGTTCAACTTTTCTATACAAAGTTGGTTAGCTCACTCATTACATATGTTCCAACTCGCTTAATTGCGAGTTTTTATTTCGTTTATTTCAATTAAGGTAACTAAAAAACTCCTTTTACTCGAGCCCGGGCCACCATGGGAATTCGATATCCACCTTAAGGGATCCCCTGATAATTGCCAACACAATTAACATCTCAATCAAGGTAAATGCTTTTTGCTTTTTTTGCGCAACTTTGTATAATAAAGTTGAACGAGAAACGTAAAATGATATAAATATCAATATATTAAATTAGATTTTGCATAAAAAACAGACTACATAATACTGTAAAACACAACATATCCAGTCACTATGAATCAACTACTTAGAAAGCTTGCTAGC

pIDT-R4mcsR3 (R4PB)

AGATCTGCTAGCAAGCTTGGGCCCCTACAGGTCACTAATACCATCTAAGTAGTTGATTCATAGTGACTGGATATGTTGTGTTTTACAGTATTATGTAGTCTGTTTTTTATGCAAAATCTAATTTAATATATTGATATTTATATCATTTTACGTTTCTCGTTCAACTTTTCTATACAAAGTTGGTTAGCTCACTCATTACATATGGCATGTTCCCCAAAGCGATACCACTTGAAGCAGTGGTACTGCTTGTGGGTACACTCTGCGGGTGCTCGAGCCCGGGCCACCATGGGAATTCGATATCCACCTTAAGGGATCCGCGCCCAAGTTGCCCATGACCATGCCGAAGCAGTGGTAGAAGGGCACCGGCAGACACGCAACTTTGTATAATAAAGTTGAACGAGAAACGTAAAATGATATAAATATCAATATATTAAATTAGATTTTGCATAAAAAACAGACTACATAATACTGTAAAACACAACATATCCAGTCACTATGAATCAACTACTTAGAAAGCTTGCTAGC

pIDT-R4mcsR3 (Bxb1PB)

AGATCTGCTAGCAAGCTTGGGCCCCTACAGGTCACTAATACCATCTAAGTAGTTGATTCATAGTGACTGGATATGTTGTGTTTTACAGTATTATGTAGTCTGTTTTTTATGCAAAATCTAATTTAATATATTGATATTTATATCATTTTACGTTTCTCGTTCAACTTTTCTATACAAAGTTGGTTAGCTCACTCATTACATATGTATGGCCGTGATGACCTGTGTCTTCGTGGTTTGTCTGGTCAACCACCGCGGTCTCAGTGGTGTACGGTACAAACCCACTCGAGCCCGGGCCACCATGGGAATTCGATATCCACCTTAAGGGATCCTGGCCGTGGCCGTGCTCGTCCTCGTCGGCCGGCTTGTCGACGACGGCGGTCTCCGTCGTCAGGATCATCCGGGCCACGCAACTTTGTATAATAAAGTTGAACGAGAAACGTAAAATGATATAAATATCAATATATTAAATTAGATTTTGCATAAAAAACAGACTACATAATACTGTAAAACACAACATATCCAGTCACTATGAATCAACTACTTAGAAAGCTTGCTAGC

pIDT-L3mcsL2

AGATCTGCTAGCAAGCTTCTCGGGCCCCAAATAATGATTTTATTTTGACTGATAGTGACCTGTTCGTTGCAACAAATTGATGAGCAATGCTTTTTTATAATGCCAACTTTGTATAATAAAGTTGGTTAGCTCACTCTCGAGCCCGGGCCACCATGGGAATTCGATATCCACCTTAAGGGATCCCAGCTTTCTTGTACAAAGTTGGCATTATAAGAAAGCATTGCTTATCAATTTGTTGCAACGAACAGGTCACTATCAGTCAAAATAAAATCATTATTTGCCATCCAGAAGCTTGCTAGC

Supplemental Figure S2. Nucleotide sequences of synthesized DNA fragments that were inserted into the donor vectors shown in Figure S1. Underlined sequences indicate multiple cloning sites.
